# The Morbid Anatomy of the Coverings of the Brain

**Published:** 1850-10-01

**Authors:** Holmes Coote

**Affiliations:** DEMONSTRATOR OF ANATOMY AT ST. BARTHOLOMEW'S HOSPITAL


### THE MORBID ANATOMY OF THE COVERINGS OF THE BRAIN.

BY HOLMES COOTE, F.R.C.S., ETC., DEMONSTRATOR OF ANATOMY AT ST. BARTHOLOMEW'S HOSPITAL.

Throughout the vertebrate kingdom the manifestation of intellect is invariably associated with the development of that portion of the encephalon known as the cerebral hemispheres: in man these masses far exceed in size any of the other segments of the cerebro-spinal centre, presenting a convoluted surface, and extending forward over the olfactory ganglia, and backwards over the cerebellum. When, as in some forms of cretinism, the hemispheres are disproportionately small, the mind is feeble and of limited capacity: deficiencies even of particular parts, such as the corpus callosum, exercise a most prejudicial influence upon the understanding. Whoever has had the opportunity of witnessing the post-mortem examinations conducted in a large asylum for the insane, cannot fail to be struck with the fact that it is in the cerebrum, or in its coverings, that morbid changes, some of uncommon character, are almost constantly met with. It becomes, therefore, the duty of all who are engaged in the study of insanity, instead of entering upon profitless discussions as to whether madness be a disease of the mind or of the body, to collect with care such observations as have been made upon the dead, to analyse their true value, and to arrange them according to their signification.

There are many morbid appearances, which, though they cannot be regarded as the cause of insanity, yet indicate very clearly a disturbed state of the cerebral circulation, upon which the mental derangement may have depended: such are opacity of the arachnoid, infiltration of the pia mater, &c. There are others, whose occurrence has exercised a direct influence upon the mind: such are softening and disorganization of some part of the brain, effusion of blood, &c. It is obvious that these two classes of morbid appearances should be arranged under different heads. Other organs, exercising an indirect influence upon the brain, require investigation: disease may have existed in the abdominal viscera which prepare the chyle; or in the contents of the chest, where the blood is arterialised and then propelled over the body. We hear it argued that post-mortem examinations of the bodies of the insane are unsatisfactory, because the same morbid appearances are met with in those who have never shown signs of mental derangement during life. To this it may be replied that the re-action of the brain to any stimulus or excitement is not the same in all people: those in full vigour will bear with a seeming impunity, what others, of weaker frame, would sink under. Many individuals endowed with just sufficient talents and strength of mind to perform the ordinary duties of life quietly and respectably, become at once unnerved, and then completely paralysed, when any sudden incident or unexpected calamity befals them. The brain of one person will bear a much greater amount of vascular excitement than the brain of another; but if the excitement were continued, the result would eventually be the same in each, and the morbid appearances observed after death would be similar. Moreover, we should be very cautious in quoting any case to prove that morbid appearances may be found in the brain of one whose mind has been sound, unless we possess an intimate acquaintance with the private and domestic character of the person. In society, we daily meet with those who, with sufficient sense and self-control to keep themselves well before the world, are nevertheless a perfect nuisance to all who have the misfortune to be closely connected with them. The following case of the kind came under my notice not long ago.

I was requested to examine the body of a gentleman, aged sixty-three, a tall, athletic man, who died quite suddenly, having been in apparent good health up to a few hours of his death. A near relative informed me that he had been a free liver, but not a drunkard; that he was a man of great sternness of character and fixity of purpose; that through life he had been violent and extreme in politics: he had been twice married and had a numerous family. On the night of November 24, 1847, he retired to rest, after having taken his usual meals: he slept longer than usual the following morning, then arose, and went to the water-closet, where he was heard groaning, as if in pain. A few minutes afterwards, he entered the sitting-room, pale and haggard, and throwing himself upon a sofa, declared that he felt stricken with death. In two hours he expired. The body was examined forty-eight hours after death. The skull-cap was thick, heavy, and full of blood; much blood escaped from the incision of the scalp; the dura mater adhered firmly to the bone; the arachnoid membrane was dry, thickened, and almost universally opaque; the layers of the pia mater were infiltrated by a large quantity of red-coloured sero-sanguineous fluid; the cerebral substance presented upon its cut surface numerous red points; the ventricles were distended by about three ounces of limpid fluid; the veins, both on the surface and at the base of the brain, were gorged and distended by black blood: there were no other morbid appearances worth recording in the other viscera. Upon my mentioning that the state of the brain here met with was similar in every respect to that observed in a class of cases common in Bethlem Hospital, I was first informed that this gentleman had never shown any sign of insanity; that he was a shrewd person, of business habits, and of active and well-regulated mind: at my request, however, the widow was questioned, and she at once stated, that for years he had been subject, without obvious cause, to fits of the most uncontrollable passion: that sometimes, in the middle of the night, he had driven her from the bedroom, and locked her out. She expressly added—"He was not intoxicated at those times." Similar cases will recur to the memory of almost any one long engaged in practice; and it may be remarked, that this excitability seems in some families to be an hereditary infirmity, which, though capable of being increased by habitual indulgence, or subdued by resolute self-control, has a tendency to manifest itself at some particular period of life, generally after the mind has been tried by business, anxiety, or grief.

I propose, in the following pages, to treat of the different organs and structures most commonly found in a morbid condition in the bodies of those who die insane. My information is gained almost exclusively from dissections at Bethlem Hospital, conducted by Mr. Lawrence, who, for several years, has very kindly permitted me to be present at these inspections; and on the present occasion has allowed me to make use of the valuable series of observations which he has collected and recorded.

Morbid appearances in the head of those who die insane have been observed not only in the substance of the brain, but also in its membranes, in the cranial bones, and in the scalp.

The vessels of the scalp may be either loaded and distended with blood, or quite empty; and this condition of the external parts may, or may not, bear any relation to the state of the parts within the skull.

When the vessels of the *scalp* are turgid, the head looks of a dusky red, or bluish hue; and this appearance often extends to the face, the features of which seem heavy, dark, and swollen. When the scalp is divided, blood flows freely; and upon its under surface small undefined extravasations are occasionally seen. When the scalp is anæmic, the integument is pallid, has a leathery feel, and the features often look shrunk and pinched.

Case I. *January* 29, 1838.—Vessels of the head, both internal and external, loaded with blood, of which a large quantity flowed out during the examination; numerous large and bloody points observed everywhere in cutting the substance of the brain. About three ounces of limpid fluid in the ventricles; convolutions flattened, as if they had been subjected to pressure: pineal body converted into a cyst, the size of a large pea, containing watery fluid.

Case II. *October* 8, 1841.—Exterior vessels of the head completely empty, and not a drop of blood escaped on making the external incisions. All the internal vessels extremely turgid; a little fluid between the dura and pia mater; a thin layer of coagulated blood, about an ounce in quantity, covered nearly the whole of the left hemisphere; but no ruptured vessel could be discovered; slight infiltration of the pia mater; vessels in the substance of the brain everywhere strongly injected; other organs healthy.

Case III. *July* 1, 1847.—The external vessels of the head were quite empty; the internal contained but little blood; the skull-cap was thick and heavy; thickening and opacity of the arachnoid over the cerebral hemispheres; considerable infiltration of the pia mater; some drachms of fluid in each lateral ventricle; and a considerable quantity remaining in the base of the skull after the removal of the brain.

The left lung filled throughout with tubercles, amongst which were several small cavities, containing pus. The lung was connected to the chest through its whole surface by adhesions, principally recent; the right lung partially tuberculated and adherent.

Out of sixty-four consecutive cases, great turgidity of the vessels of the scalp was noticed eleven times; the contrary, or anæmic condition, not more than three or four times.

The *cranium* does not present in the insane any anomalies of form worthy of notice, except that in a considerable number of cases the skull-cap is shallow, especially in the frontal region. There are, however, great varieties in the thickness, consistence, and weight of the bone. The normal skull-cap is rarely of the same thickness throughout, being thinnest and often translucent in the temporal region. If a strong part be selected—namely, that part of the occipital bone which corresponds with the lateral sinus, the measurement in the insane will be found to vary from one-eighth to five-eighths of an inch. The weight depends, cæteris paribus, upon the density of the bone. A thick, dry skull-cap, of medium size, about three-eighths of an inch in its thickest part, will weigh 6950 grains; a skull-cap of average density in structure and the same thickness, 6200 grains. A very dense skull-cap, weighed shortly after removal from the body, 7950 grains. A shallow, porous skull-cap five-eighths of an inch in thickness, weighed only 4800 grains. It is easy to see with the naked eye the difference in the density of the bone; the cancellous diploe being in some instances well marked; in others entirely obliterated.

The most common cause of this thickening and hypertrophy of the bone is chronic inflammatory action going on for some time before death; the vessels of the bone become filled with blood, so that upon tearing away the dura mater and the pericranium, which in these cases usually adhere very firmly, the whole skull-cup appears of a pink colour, produced by the injected vessels, which are distinctly visible with a magnifying glass. It not uncommonly happens that an irregular growth of bone takes place at some part of the skull, generally on the inner surface of the frontal bone, from whence the dura mater becomes thinned and adherent to subjacent parts, and the cerebral hemispheres are partially compressed and flattened. Rokitansky remarks that this form of "hyperostosis" of the skull arises from oft-repeated attacks of acute, or from a continued attack of chronic inflammation, in which the vessels of the pericranium or of the dura mater may take part.

Case IV. *March* 4, 1844. The skull-cap was heavy; the bone, where it was thickest, and the diploe most abundant, had a livid colour, from turgidity of the blood-vessels. The dura mater adhered to the bone so firmly, that it was difficult to detach the skull-cap, and the membrane was considerably torn in accomplishing this operation. The arachnoid covering the hemispheres was partially opaque, and there was considerable serous infiltration of the pia mater.

Case V. *August* 8, 1845.—The skull-cap was extremely heavy and thick; the sutures were almost entirely obliterated. From the inner surface, near to the crista galli process of the frontal bone, there projected from a surface equal in extent to a crown piece, a number of smooth, rounded eminences, to which the dura mater was firmly adherent.

Case VI. *March* 13, 1848.—There was a large ulcerated cancer in the right breast. The subcutaneous cellular tissue was anasarcous over the whole body, and there was a considerable quantity of dropsical fluid in the abdomen and left pleura.

The entire skull-cap was of preternatural thickness throughout; in the frontal and occipital regions it measured three-quarters of an inch. The bone was very vascular, and of less compact structure than in the normal state. The vessels of the dura mater were turgid.

Case VII. *June* 30, 1845.—The frontal bone, to an extent of about an inch square on each side of the middle line, was twice the regular thickness. Nothing abnormal was observed on the external surface of the bone, but the internal table presented a very irregular surface, raised into prominences not exceeding the size of a large pea, fissured and excavated at one part, so as to make the bone preternaturally thin. The dura mater in this situation was not increased in thickness, nor otherwise altered, but it adhered so firmly, that it could not be stripped off in the usual way. The structure and consistence of the bone were perfectly natural. The thick portion presented the regular arrangement of two tables and diploe. The surface of the brain and the immediately investing membranes, opposite to this portion of the frontal bone, was quite natural. This state of the bone must have been of very long standing, if not an original formation; and, in either case, could have had no connexion with the mental disorder. The blood-vessels of the membranes and brain, both on the surface and throughout the substance of the organ, were turgid. The arachnoid was partially opaque on the convexities of the cerebral hemispheres, and the pia mater was infiltrated in the same situation.

Acute inflammation of the *dura mater* is extremely rare, except as a result of injury; but chronic inflammation, producing thickening of the membrane, or close adhesion to the neighbouring bone, is of common occurrence. In these cases there is effused between the membrane and the bone which participates in the inflammation, a layer of lymph, which soon becomes organised. As has been already mentioned, so close an union is established between these parts, that upon tearing away the skull-cap, an operation which requires considerable force, shreds of the dura mater often come with it. This preternatural adhesion of the dura mater may occur about eight or ten times in every fifty cases.

It sometimes happens, where the vessels of the head are very full, that an extravasation of blood takes place between the dura mater and the bone. In one case (VIII.) which presented this morbid condition, there was also hypertrophy of the left ventricle of the heart.

Case IX.—About half a teaspoonful of coagulated blood was found between the cranium and the dura mater, on the left side of the longitudinal sinus near the parietal bone. All the blood-vessels of the brain and its membranes were gorged to the utmost extent. Bloody points extremely numerous wherever the cerebral substance was cut, arachnoid coat thickened and opaque. The left ventricle of the heart was thick, firm, and partially hypertrophied.

Neither ossification of the folds of the dura mater, nor layers of bony structure, produced by the organisation and subsequent ossification of effused lymph, are peculiar to the insane. Sometimes we meet with fibrous growths, which, springing from the exterior of the dura mater, make their way into the bone by causing its gradual absorption, until partial perforation ensues.

Case X. *September* 25, 1848.—The bones of the cranium were thick and dense, so that the skull-cap was very heavy. The frontal bone presented on its left side, midway between the orbit and the coronal suture, an irregularity of surface, to an extent equal to that of a crown piece. There was a central depression, with superficial furrows leading to it. The internal table presented corresponding appearances, with greater roughness. In the centre of the depression the bone was quite thin, so as to transmit light. The dura mater adhered very firmly in this situation, being generally thickened, and having a small fibrous tumour which filled up the excavation of the bone growing from it. The pericranium over the part now described was in its normal state, and neither the bone nor its external coverings presented any appearance of active disease. The visceral surface of the dura mater, beneath the fibrous growth, was strongly adherent to the cerebral arachnoid, over a space of nearly two inches square. The arachnoid covering the hemispheres was partially opaque, and the pia mater was infiltrated in the same situation.*

Both tubercle and cancer of the dura mater are uncommon diseases, and do not seem to bear any relation, from the rarity of their occurrence, with any of the ordinary forms of insanity. A hospital for the insane is not likely to afford any considerable number of cases of cancer, but tuberculosis, often in its most aggravated forms, involving not only the thoracic, but also the abdominal viscera, is one of the most usual complications. I cannot, however, recal an instance of tuberculosis of the dura mater; it being as uncommon in this situation as in the other fibrous structures. Rokitansky believes that the deposit, when found adhering to the inner surface of the dura mater, has been originally effused on the cortical substance of the brain, from whence being separated it has contracted this secondary adhesion.

Although the *arachnoid membrane*, in common with all other serous membranes, is usually described as a shut sac, it must not be imagined that the vascular layer, which is extended over the cerebral hemispheres, is prolonged over the dura mater. Diseases of the parietal portion, which are rare, should more properly be considered as diseases of the inner surface of the dura mater. Diseases of the visceral portion, which are common, are usually, but not always, associated with disease of the pia mater, so that after death both these structures present morbid appearances.

Upon the parietal layer of the arachnoid, *i. e.*, upon the inner free surface of the dura mater, we not uncommonly meet with adventitious membranes, which have been considered by several authors as originating in extravasated blood, which, spread out in a thin layer over the surface of the hemispheres, loses its colouring matter, and then becomes organised. For a description of these structures I refer to the cases related below; but I will here state the grounds upon which the opinion of their hæmorrhagic origin has been formed.

I. The absence of any inflammatory change of structure in the arachnoid, especially in the parietal portion to which the adventitious structure is attached.

II. That in the recent state the effusion appears as pure blood.

III. The peculiar change of structure which the extravasated fibrin undergoes, and the absence of an intimate organic connexion with the dura mater.

As these reasons are commonly considered conclusive, I will relate some cases, which will show, better than any argument, how far they may be trusted.

1. The dura mater, and commonly also the visceral arachnoid, exhibit in these cases signs of long continued inflammatory action.

Case XI. *Jan.* 29, 1849.—The blood vessels of the dura mater were turgid. On cutting through the membrane, a considerable quantity of reddish fluid escaped. The *entire internal* surface of the dura mater was lined with a continuous adventitious production of red colour, varying thickness, softish consistence, so as to be easily lacerated, adherent externally, but having a free and smooth internal surface, like that of a serous membrane. It was thinnest on the base of the skull, and the superior surface of the tentorium, thicker over the cerebral hemispheres, but thickest over the falx, as well as over the extremities of the anterior and posterior lobes. The thinner part was nearly transparent and colourless when stretched out; but when folded together, it was of decided red colour. It was about the thickness of the arachnoid, as seen at the base of the brain separate from the pia mater, and at some parts approached in firmness to that membrane. The thicker portions were perfectly opaque, nearly as thick as the mucous membrane of the bladder, of a deep, dull red colour, here and there as dark as that of coagulated venous blood, and presenting an appearance of ecchymosis, which appearance was also observable in some situations where the structure was thin. On the anterior and outer part of the right hemisphere, there was an irregular cavity between layers of the adventitious production, not well defined, nor with smooth sur-

* A very interesting case of a fibrous tumour growing from the dura mater lining the left orbital fossa of the frontal bone, and apparently produced by external violence, is recorded by Dr. John Lyell, in the September number of the Edinburgh Monthly Journal.

face; it contained some of the reddish fluid already mentioned. The arachnoid membrane was much thickened, and completely opaque along the great longitudinal fissure of the cerebrum; and a similar change, in slighter degree, extended over the whole cerebral hemispheres. The cellular texture of the pia mater was infiltrated. The blood vessels of the cerebrum were turgid, particularly those in the cerebral substance. There was about an ounce of limpid fluid in each lateral ventricle; much fluid, discoloured by blood, remained in the base of the skull after the brain had been removed.

The above may be taken as a fair specimen of the class of cases now under consideration. Increased vascularity, and morbid changes of structure, the result of inflammation, were noticed in almost all the parts contained within the cranium.

2. We may regard that part of the effusion as most recent, which is most semifluid, or softest, and the least firmly connected to the membrane on which it lies; and this, so far from being pure blood, is very often a substance of yellowish white colour, which closely resembles a layer of lymph as effused in the pleural or peritoneal sac. In one instance examined in Bethlem, this effusion had formed at the base of the skull only, the upper surface being covered by purulent fluid. There were some extravasations of blood also at the base of the brain, but they were limited in extent, and quite distinct from the adventitious membrane.

Case XII. *March* 22, 1849.—Skull-cap extremely thin and light; arachnoid membrane dry; over the entire surface of both cerebrum and cerebellum the pia mater was infiltrated by a quantity of yellowish green purulent fluid, which dipped down between the convolutions, and followed the reflexions of the membrane in every part. At the base of the skull a thin layer of lymph, effused into the sac of the arachnoid, was spread over the basilar process to the occipital fossa of the occipital bone. The contiguous margins of the brain at the fissure of Sylvius were adherent, and there were three or four superficial extravasations of blood, the largest of which pursued the course of the olfactory nerves, lying between the thickened arachnoid and the grey vesicular cerebral substance. The brain, though more vascular than natural, was otherwise sound. The ventricles contained about three ounces of turbid yellow, purulent fluid.

3. The soft semifluid effusion undergoes no peculiar transformation. On the conversion of its elements into a continuous membrane, a mass of globules speedily coalesce, by elongation, into flat fibres, in many respects resembling the fibre formed by the coagulation of fibrin. The membrane, thus constituted, contracts a most intimate connexion with the adjacent dura mater. Vessels of minute size, but infinite in number, filled without much difficulty by injection from the middle meningeal artery, enter the adventitious structure, and ramify after a determinate arrangement. It is a very great error to suppose in these cases, either that the dura mater is unchanged in structure, or that the adventitious membrane is not intimately connected with it. I have, with far greater ease, separated from the surface of the liver a continuous layer of lymph, the product of an attack of acute peritonitis.

The true character of these membranes is described by Rokitansky,* under the head of "inflammation of the arachnoid." He remarks that—

"The inner surface of the dura mater appears strongly injected, and of rosy colour, and covered by a layer resembling mucus, or of firmer consistence, like coagulated fibrin, of yellowish colour, and purulent in character." Yet still, in this country, all such effusions are indiscriminately pronounced to be hæmorrhagic, notwithstanding that there are abundant cases on record which prove, that where blood is effused in any quantity into the arachnoid sac, it does not spread equally over the convex surface of the hemispheres, but accumulates in larger quantity about the spot from whence the blood flowed. These points, readily observable in the upper part of the brain, are yet more clearly manifested in the examination of effusions at the base of the skull.

Inflammation of the *visceral arachnoid* may be either acute or chronic. The morbid changes extend very frequently, but by no means invariably, to the subjacent pia mater. Acute arachnitis is most commonly met with as a consequence of violence, or some change of structure in the cranial bones. As a primary affection it is rare: its effects are well illustrated in the following case:—

Case XIII. *March* 30, 1847.—The blood vessels of the cranial coverings, of the cranium, and of the dura mater, quite empty.

---

* Pathologishe Anatomie. Book ii., § 718.

Acute inflammation of the arachnoid over the whole surface of the cerebrum, cerebellum, and medulla oblongata. These parts, and the internal surface of the dura mater plentifully smeared with a thickish, yellow, puriform fluid. The minute vessels of the pia mater injected, and the membrane generally of a pink tint over the cerebral convolutions. Partial infiltration of pus in the pia mater, particularly on each side of the great fissure, mostly at the surface, but descending at a few points for a short distance between the cerebral convolutions. Thickening of the arachnoid, and purulent infiltration under it, at the middle of the base of the brain. Thickening and yellow colour of the velum interpositum, extending to the edges of the choroid plexus; the fluid in the lateral ventricles not much beyond the natural quantity, turbid and yellowish. These morbid appearances were observed but once in ninety consecutive cases.

Chronic inflammation of the arachnoid produces morbid changes, which are very frequently met with in the examination of the bodies of the insane. They are seen also, though to a less extent, in those who, during life, have been addicted to drinking, or to any habit by which the cerebral circulation has been habitually excited. In the earliest stage, the secretion is arrested, and the membrane, when its sac is opened, appears preternaturally dry; next its transparency is lost; a milky discoloration pervades the whole, or parts, of the surface; frequently numerous opaque points and dots are seen studded over those parts where the congestion has been greatest. Thickening then ensues; the membrane becomes converted into a dense, perfectly opaque white structure, through which the subjacent hemispheres can be seen with difficulty.

Sometimes, though more rarely, there is an increased quantity of fluid in the arachnoid sac. It may be serous, fibrinous, or bloody. It is sometimes stained with bile in the bodies of those who die jaundiced. Arachnitis, either acute or chronic, is generally combined with inflammation of the pia mater; but morbid changes are more frequently seen in the latter than in the former membrane. Out of 60 consecutive cases, morbid appearances were observed 26 times in the arachnoid membrane, and 54 times in the pia mater. Of the 28 instances of diseased arachnoid, there were recorded—Preternatural dryness, 3; Simple congestion, 1; Effusion into the sac, 2; Thickening and opacity, 22. A thin film of coagulated blood has sometimes been found in the arachnoid sac covering the convexity of the hemispheres to a greater or less extent. Its character has always been perfectly well marked, and it differs in a way not to be mistaken from the inflammatory effusions on the inner surface of the dura mater. In these cases, the neighbouring structures are usually unaltered in appearance, the dura mater is but loosely attached to the skull-cap; the arachnoid membrane preserves its transparency; the blood, easily separable from the part on which it lies, is thickest about the spot from whence the hæmorrhage proceeded, where also the red colour is deepest.

Tuberculous deposit in the arachnoid membrane is rare.

The *Glandulæ Pacchioni*, small granular bodies, composed of condensed areolar tissue, found mostly along the course of the superior longitudinal sinus, are described, I believe incorrectly, amongst the morbid changes of the arachnoid. They exist at most ages, but are far more numerous in the old than the young. They are not confined to the arachnoid cavity, being seen within the longitudinal sinus, and on the outer surface of the dura mater, next to the bone, which is often hollowed out to receive them. Although their number varies considerably, they are so constantly present in the crania of all subjects, that it appears to me further evidence is required before we can class them amongst morbid products. The use of these bodies is unknown.

The *pia mater*, the vascular membrane of the brain, is, as might be expected, usually found altered in structure in all cases where the functions of the cerebrum have been disturbed, either by simple congestion or by inflammation. The state of active or passive congestion is seldom seen, patients rarely dying until that stage is passed, and has been succeeded by alterations of structure of more permanent character. When the opportunity occurs of examining the pia mater in a state of active congestion, we find the whole surface of the cerebrum covered by a close net-work of anastomosing blood-vessels, forming polygonal spaces of small size, and giving off at right angles minute branches, which pass directly into the grey covering of the hemispheres. The pia mater is usually closely applied to the cerebral substance, no effusion separating the two. Passive congestion, often connected with a diseased condition of the heart or lungs, is indicated by fulness of the large venous trunks on the surface of the brain: the blood is of dark colour, and the vessels of the pia mater, though often distended to their minuter ramifications, want that bright red, highly-injected appearance characteristic of active arterial congestion. I have seen both conditions: the one in cases where death has ensued from apoplexy; the other, where it has been produced by slow suffocation.

Serous infiltration of the pia mater (called by Rokitansky, œdema) is the commonest morbid appearance observed in the insane. Out of sixty cases, it was recorded forty-six times, exclusive of those instances in which the effusion was a consequence of the gradual atrophy of the brain. The layers of the pia mater become separated from one another; it loses its membranous structure, and appears a spongy tissue, several lines, or even a quarter of an inch, in thickness. The fluid is either of watery or jelly-like consistence, and the blood-vessels, tortuous and often distended, may be readily seen meandering through it. The whole convex surface of the hemispheres, and the fissures between the convolutions, are usually equally covered by the diseased structure; it rarely is confined to any one part of the encephalon.

Case XIV. *December* 19, 1848.—The vessels of the cranial contents generally turgid, and the bloody points in the cerebral substance conspicuous. Great infiltration of the pia mater, so that it constituted a covering not less than one-third of an inch in thickness over the whole convexities of the cerebral hemispheres. The fluid of the lateral ventricles was somewhat increased in quantity, and slightly turbid. The left ventricle of the heart was thick and firm.

In simple congestion of the pia mater, the fluid effused between its layers is of watery consistence, and contains a small quantity of albumen in solution. When inflammation attacks the membrane, the fluid is either thick and jelly-like, loosely coagulated, and contains fibrin, or it is purulent, and of yellowish green colour. The disease being uncommon, I will mention the particulars of some cases. I may refer, however, to Cases XII. and XIII., which show that the disease is often limited to the deep surface of the pia mater, the contiguous cerebral substance presenting no corresponding changes worthy of notice. The membrane may then be readily stripped off. Other cases, however, occur in which the disease is not thus limited; the adhesion between the pia mater and the cerebral substance being so intimate, that fragments of the convolutions are torn off in attempting to effect a separation. The deeper structures of the cerebrum may also be involved, contrary to the opinion of Rokitansky, who affirms that in this form of inflammation, "the brain itself, with the exception of the cortical substance, does not sympathize."—"Das Gehirn selbst verhält sich—etwa mit Ausnahme des Gehirnzinde—indifferent."*

Case XV. *August* 18, 1845.—The body emaciated. The skull thin; not much blood in its vessels, or in those of the dura mater. The surface of the arachnoid dry. The brain turgid, so that it bulged somewhat over the cut edge of the cranium, when the dura-mater had been cut round. The arachnoid thickened, and slightly opaque over the cerebral hemispheres, and at the base of the brain, particularly along the middle line of the organ, including the optic commissure and tracts, the infundibulum pons, and the commencement of the chord. Slight serous infiltration of the subarachnoid tissue of the hemispheres.

Yellow discoloration of the surface was observed in portions of small extent, about six or eight in number, on the cerebral hemispheres, particularly on the flat surfaces forming the sides of the longitudinal fissure. This appearance was found to be produced by interstitial deposition (of fibrin?) into the pia mater, rendering it thick and firm, and extending to the processes between the convolutions. These thickened portions adhered so firmly, that the cerebral substance was lacerated in detaching them. This change had taken place to the greatest extent in the pia mater covering the corpus callosum, where it was converted into a thick, tough, yellowish mass, adhering most firmly to the inferior edges of the hemispheres, and to the corpus callosum. The cerebral substance, which was torn away in detaching the diseased membrane, was itself reddened and filled with small portions of coagulated blood, so as to present an ecchymosed appearance, to the depth of a quarter of an inch. The lateral ventricles contained about three ounces of slightly turbid fluid. The roof of these cavities, the corpus callosum, the septum lucidum and fornix, were softened almost to the consistence of thick cream, but retained their natural white colour. This softening was a morbid

* Pathologishe Anatomie. Book ii. § 729.

change, not produced by decomposition; for there was no discoloration nor bad smell, and the rest of the brain had its natural firmness.

There was extensive tuberculous disease of both lungs.

There is no instance in the collection of cases which form the basis of this report of primary cancer of the pia mater. Tuberculosis, also, is not common. According to Rokitansky the deposit is first effused on the surface of the cerebrum, and then, becoming embedded in the pia mater, contracts with it secondary adhesions. Commencing at the base of the brain, it gradually extends to the upper convex surface, and gives rise to inflammatory attacks of various degrees of intensity. Upon referring to the series of cases from which the preceding observations have been taken, I do not find that mention is made of the presence of this deposit in the membranes, even in those cases where the tuberculous diathesis has been most strongly marked, and where the viscera have been found most extensively diseased. The following is a good instance of the kind:—

Case XVI. *June* 5, 1848.—A few opaque white spots were observed upon the arachnoid; the pia mater was infiltrated by serous fluid; the cerebral substance was firm and bloodless. The opposed surfaces of both pleuræ were united by old adhesions. Masses of grey semi-transparent tubercle, varying in size from a pin's-head to a large bean, were deposited throughout the substance of the lung, being especially numerous in the upper lobes, where there were numerous cavities. The sac of the pericardium was obliterated by old adhesions. A considerable quantity of yellow serous fluid was collected in the sac of the peritoneum. Tuberculous matter was deposited in the mesenteric glands, and in the substance of the pancreas; also in the absorbent glands accompanying the large vessels, and round about the cæcum. There were numerous ulcers of circular form in the ileum and the commencement of the large intestine. The viscera were firmly adherent to the posterior part of the abdominal cavity.

From the evidence here collected, we may infer that by far the most frequent morbid condition of the coverings of the brain in cases of insanity is one of *increased vascularity*, and that the traces of this disturbance are the more commonly met with, and most clearly marked, the nearer we approach that most vascular structure (the pia mater) which is composed of minute arteries, so arranged as to pass directly into the cerebral substance. The vessels, both of the scalp and of the cranium, may be likewise turgid; an opposite, or anæmic condition, however, occasionally presents itself, which is not at all incompatible with fulness of the vessels within the skull. But all cases of insanity cannot be classed in this division, for instances are met with, in which the vessels, both on the exterior and in the interior of the head are empty, or in which there is so slight a deviation from the normal state, that the organ might be pronounced healthy. In these cases we are called upon to investigate the minute structure of the cerebral substance, the quality of the nutrient fluid by which it is supplied, and the state of the organs of digestion, respiration, and circulation.

